# Angiotensin-converting enzyme inhibitory peptides and isoflavonoids from soybean [*Glycine max* (L.) Merr.]

**DOI:** 10.3389/fnut.2022.1068388

**Published:** 2022-11-24

**Authors:** Ayyagari Ramlal, Aparna Nautiyal, Pooja Baweja, Vikash Kumar, Sahil Mehta, Rohit Kumar Mahto, Shikha Tripathi, Aravindam Shanmugam, Bingi Pujari Mallikarjuna, Pushpa Raman, S. K. Lal, Dhandapani Raju, Ambika Rajendran

**Affiliations:** ^1^Division of Genetics, Indian Council of Agricultural Research (ICAR)-Indian Agricultural Research Institute (IARI), New Delhi, India; ^2^Department of Botany, Deshbandhu College, University of Delhi, New Delhi, India; ^3^Department of Botany, Maitreyi College, University of Delhi, New Delhi, India; ^4^Faculty of Agricultural Sciences, Institute of Applied Sciences and Humanities, GLA University, Mathura, Uttar Pradesh, India; ^5^Department of Botany, Hansraj College, University of Delhi, New Delhi, India; ^6^School of Biotechnology, Institute of Science, Banaras Hindu University (BHU), Varanasi, Uttar Pradesh, India; ^7^Indian Council of Agricultural Research (ICAR)-National Institute for Plant Biotechnology (NIPB), New Delhi, India; ^8^Department of Botany, Institute of Science, Banaras Hindu University (BHU), Varanasi, Uttar Pradesh, India; ^9^Centre for Plant Breeding and Genetics, Tamil Nadu Agricultural University, Coimbatore, Tamil Nadu, India; ^10^Division of Genetics, Indian Council of Agricultural Research (ICAR)-Indian Agricultural Research Institute (IARI), Regional Research Centre, Dharwad, Karnataka, India; ^11^Department of Plant Breeding and Genetics, Tamil Nadu Rice Research Institute, Tamil Nadu Agricultural University, Aduthurai, Tamil Nadu, India; ^12^Division of Plant Physiology, Indian Council of Agricultural Research (ICAR)-Indian Agricultural Research Institute, New Delhi, India

**Keywords:** soy products, angiotensin-converting enzyme I (ACE I), cardiovascular diseases (CVDs), natural drugs, renin-angiotensin-aldosterone system (RAAS)

## Abstract

Angiotensin-converting enzyme I (ACE I) is a zinc-containing metallopeptidase involved in the renin-angiotensin system (RAAS) that helps in the regulation of hypertension and maintains fluid balance otherwise, which results in cardiovascular diseases (CVDs). One of the leading reasons of global deaths is due to CVDs. RAAS also plays a central role in maintaining homeostasis of the CV system. The commercial drugs available to treat CVDs possess several fatal side effects. Hence, phytochemicals like peptides having plant-based origin should be explored and utilized as alternative therapies. Soybean is an important leguminous crop that simultaneously possesses medicinal properties. Soybean extracts are used in many drug formulations for treating diabetes and other disorders and ailments. Soy proteins and its edible products such as tofu have shown potential inhibitory activity against ACE. Thus, this review briefly describes various soy proteins and products that can be used to inhibit ACE thereby providing new scope for the identification of potential candidates that can help in the design of safer and natural treatments for CVDs.

## Graphical Abstract 1

The classical RAAS summarizes the process of formation of biologically active octapeptide angiotensin II. The degradation is a two-step process wherein the renin cleaves N-terminal of Angiotensinogen (AGT) to form Angiotensin I (Ang I; 10-amino acid long and biologically inactive) and in the second step, angiotensin-converting enzyme (ACE I) cleaves Ang I to yield an octapeptide angiotensin II (biologically active). The renin is released by the JG cells when the volume of circulating blood is reduced while the AGT is constitutively released by the liver. The Ang I is acted by the ACE I to form ACE II which disrupts the synthesis of nitric oxide (NO) and it also recruits aldosterone to elevate the blood pressure. 
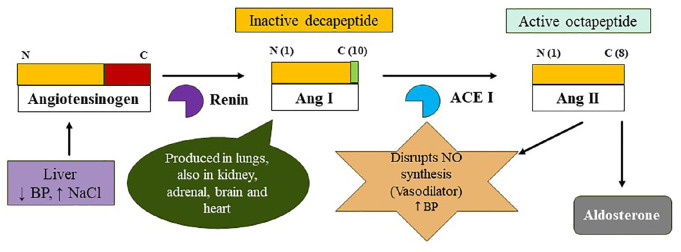

## Graphical Abstract 2

Different sources of bioactive peptides from soybean and its products. 
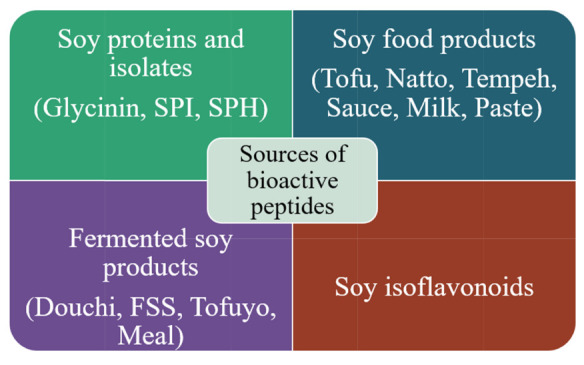

## Introduction

Cardiovascular diseases (CVDs) are leading cause of mass mortality around 17.9 million deaths each year (1). The primary reason for CVDs is hypertension which affects vital organs like the brain and kidneys. Several other pathophysiological processes also occur simultaneously which include stiffening of large ducts (aorta, carotid artery) and elastic artery, smooth muscle cell proliferation, vasoconstriction, and dysfunction of the endothelium (2). The renin-angiotensin-aldosterone system (RAAS) helps in the regulation of fluid balance and plays a crucial part in maintaining homeostasis of the cardiovascular system and normalizing blood pressure (BP) (3). One of the components of the RAAS is the angiotensin-converting enzyme (ACE). ACE was first isolated in 1956 from rat kidneys (4). It is essentially required for the regulation of the formation of angiotensin II (Ang II) from its precursor molecule angiotensin I (Ang I) and in turn, increases BP. The inhibitors aid in the regulation of blood pressure levels by inhibiting the formation of Ang II and thereby prevent CVDs (4–11). Renin is the primary enzyme involved in the RAAS and is required for the formation of Ang I and is released into the bloodstream. This enzyme cleaves a stretch of 10 amino acid residues from the N-terminal region of angiotensinogen leading to the production of Ang I. This peptide is acted upon by ACE which then forms Ang II and stimulates the release of aldosterone, which eventually elevates BP (3, 5, 9, 12). Simultaneously, the degradation of a potent vasodilator, bradykinin is also catalyzed (2). BP is a complex process involving a series of steps with the involvement of multiple organs, and the autonomic nervous system (ANS), vasopressor and vasodepressor hormones, the total volume of body fluid, renal function, and the vasculature. The endothelium is directly involved in controlling BP by producing multiple vasodilators and vasoconstrictors such as nitric oxide (NO) (2, 13, 14).

The ACE inhibitors (ACEIs) bind to ACE competitively thereby restricting the Ang I to Ang II conversion thus limiting the circulation of Ang II. There are various commercially available synthetic ACEIs are classified as carboxyl containing such as Lisinopril, Benazepril, Perindopril, Cilazapril, Quinapril, Ramipril, and trandolapril contain greater lipophilicity over other ACEIs, other group that include phosphoryl containing such as Fosinopril. These ACEIs groups have replaced the sulphydryl group containing first ACEI (Captopril) that cause skin rashes, proteinuria and disturbed taste along with several other effects including headache, hyperkalemia, nausea, fatigue, dizziness, swelling of the lower portion of the skin, cough, and angioneurotic edema (3, 15). Plant compounds have been reported to have ACE inhibitory activities as reviewed by Patten et al. (16) that do not cause such severe side effects and are safe for consumption. The plant-based bioactives are better alternatives to synthetic drugs due to their non-toxic, ease of availability, and no side effects (13, 14, 16, 17). Therefore, there is a need to switch and find alternative natural or plant-based sources, especially from food proteins (protein-derived bioactive peptides) having promising health-promoting benefits with less or no side effects (17–20). Previously, many ACEIs have been identified from legumes. For instance, Akıllıoğlu and Karakaya (21), inhibitory activity of ACE was observed using common and pinto beans and green lentil, similarly, Roy et al. (22) analyzed pea, chickpea and lentil. Boschin et al. (23) analyzed the enzymatic protein hydrolysate activity for ACE using *Lupinus albus* L., pea, lentil, chickpea, common bean, and soybean. Lentil, black soybean and black turtle bean were tested for the presence of ACE inhibitory effects by Zhang and Chang (24) using *in vitro* gastrointestinal proteolytic digestion (24) and Bollati et al. (25) used pea. Among legumes, one such wonder and multipurpose crop is the soybean.

*Glycine max* (L.) Merr. (Soybean) is one of the economically important and versatile legumes. It contains a plethora of nutritional compounds including proteins (40–42%), and lipids (15–20%) including polyunsaturated fatty acids (PUFA), alpha-linolenic acids, vitamins, and minerals (26–30). Soybean is native to East Asia, probably North and Central parts of Asia and it has been cultivated in China for 4,000 years (31). Soybean has been widely associated with reducing BP and obesity. It shows anti-cholesterol activity by lowering both genic and non-genic origin-based hypercholesterolemic and triglycerides, thus reducing the risk of CVD along with it is also reducing postmenopausal symptoms, and risk of osteoporosis and antimutagenic effects (32). It also possesses hypotensive activities like inhibition of ACE I, and anti-microbial and anti-thrombotic activities (33). Soybean extracts serve as a primary ingredient in many drug formulations curing various deadly diseases. For instance, cyanidin-3-glucoside (an anthocyanin) found in black soybean helps in treating diabetes and obesity. It has also been shown to act as an antineoplastic agent and helps in scavenging free radicals (32, 34). It possesses anti-inflammatory and anti-proliferative effects on human HT29 colon cancer cells and human leukemia Molt 4Bcells (32, 35, 36). The Bowman–Birk protease inhibitor (BBI) and its concentrate (BBIC) when administered orally lowers inflammation and demyelination of the spinal cord (37, 38) and lunasin is used as anti-inflammatory and anti-cancer peptide (39, 40). Soybean is a high proteinaceous legume, it acts as an ideal source for the identification of bioactive peptides against hypertension with other effects (10). Recently, Ramlal et al. (28) showed that soybean isoflavonoids especially Genistein can also be used against ACE (28).

The current article aims to emphasize various ACE inhibitors identified in soy proteins and fermented foods that would eventually be helpful in the identification and development of novel functional food additives and useful in the design of safer drugs for ACE inhibition.

### Angiotensin-converting enzyme I: The key player

**FIGURE 1:**
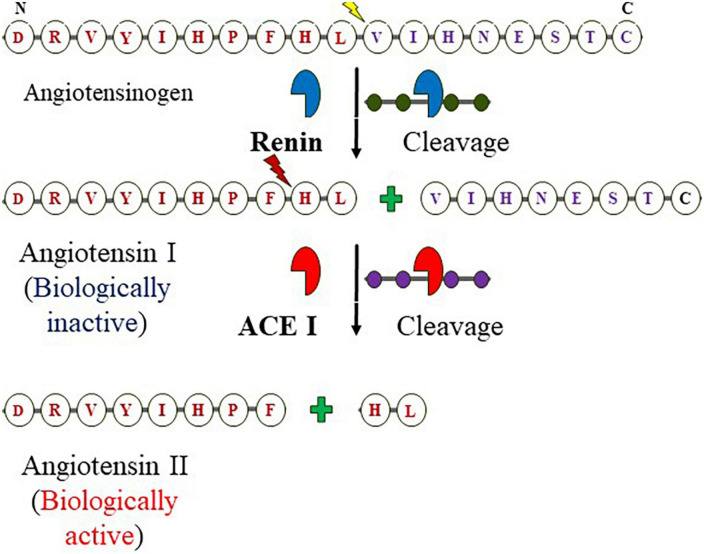
Components of RAAS and formation of angiotensin II from angiotensinogen: The enzymatic reaction cleaves off N-terminal of angiotensinogen by the kidney-derived enzyme renin at Leucine–Valine to form angiotensin I (decapeptide) which is in turn digested by the endothelium-bound angiotensin-converting enzyme (ACE I) at Phenylalanine-Histidine yields the octapeptide.

Angiotensin-converting enzyme I (ACE I) with EC 3.4.15.1 is a zinc-containing chloride-dependent peptidyl-dipeptidase. A enzyme (15, 41, 42) and helps in maintaining the homeostasis of the cardiovascular system (Figure 1) (43). Two isoforms of ACE exist; the somatic ACE (sACE) and testicular ACE (tACE) which contain one and two catalytically active domains, respectively, referred to as N and C termini in the sACE (called cACE and nACE). Although, the two domains exhibit high sequence and structural similarity, show distinct substrate specificity and inhibitor binding mechanisms (5, 44). The structure of ACE consists of 27 helices which include 20 alpha−helices and seven 3_10_ helices, and six short beta strands (42). The ACE is an important component of the RAAS system (45). RAAS is an endocrine system that balances systemic BP and maintains the balance of fluid-electrolytes (46, 47). The pathway begins in the juxtaglomerular cells (JG) as it helps in the biosynthesis of renin in the renal glomerulus. Initially, renin is an immature preprohormone (prorenin) following this bioactive is formed through the proteolytic cleavage of 43 amino acids from the N-terminal (3). The first rate-limiting step in the pathway is the N-terminal cleavage of AGT by the renin forming an inactive decapeptide Ang I or Ang (1–10). AGT is stored primarily in the liver (3, 48), it is constitutively secreted (thus plasma levels remain normal), however, its expression is also observed in other tissues like the adrenal gland, heart, brain, kidney, vascular, adipose tissue, placenta and ovary, and also in vascular endothelial cells (3, 49–51). This biologically inert decapeptide Ang I is then hydrolyzed to form octapeptide Ang II [Ang (1–8)] by the enzyme called ACE I. This octapeptide is a potent vasoconstrictor and biologically very active (3), as depicted in Figure 1. Ang II acts directly on vessels and thereby stimulates vasoconstriction leading to an increase in BP. Similarly, it also acts on adrenal glands to stimulate the release of aldosterone. The released aldosterone further acts on the kidneys to stimulate the reabsorption of water and NaCl, thereby increasing blood volume and pressure due to renal and systemic arterioles constrictions (3, 49).

### Angiotensin-converting enzyme inhibitors derived from soybean and its various products

There are many conventional and modern methods used for the prediction, identification, and discovery of biologically active peptides. The conventional approaches involve the selection of enzymes and proteins for proteolysis, fractionation, and identification followed by analysis. However, the modern or *in silico* based-approach includes virtual screening, and structure-function analysis through various computational methods followed by molecular docking studies (52). Food-derived peptides derived from functional food products are nowadays being used for the identification of novel inhibitors of ACE (53). Using the conventional and *in silico* approaches, various groups have discovered and identified many peptides showing inhibitory action against the ACE which are being discussed below.

There are many *in vitro* assays which are being used to investigate the activity of ACEIs based on the substrate used which include Cushman and Cheung Method using a substrate hippuryl-histidyl-leucine (HHL) (54), Carmel and Yaron method used substrate o-aminobenzoylglycyl-p-nitrophenylalanilproline (55), Holmquist method using a substrate furanacryloyl-tripeptide (56), Elbl and Wagner method considered benzoil-[l-14C] glicyl-L-histidine-L-leucine as a substrate (57), and Lam method using 3-hydroxybutyrylglycyl-glycyl-glycine as substrate (58). Several different methods to measure the results of enzymatic reactions or separating substrate with products, including spectrophotometric, fluorometric, high-performance liquid chromatography (HPLC), electrophoresis, and radiochemistry (59). Table 1 describes and summarizes the ACEIs so far from soybean which are mostly identified using the enzymatic processes and also through *in silico* molecular docking approaches.

**TABLE 1:** Summary of inhibitory soybean bioactive peptides with their IC_50_ values and interacting ACE subsites.

S. no.	Soy product	Bioactive peptides	Inhibitory concentration	Approach adopted	ACE binding pockets^#^	Model used	References
1	Fermented soy sauce	Hw fraction	–	Gel filtration chromatography	–	Hypertensive rats	(72)
2	Soy paste (fermented)	HHL	2.2 μg/ml	HPLC	–	–	(73)
3	Soy protein	–	100 mg/kg of body weight/day	Oral administration	–	Spontaneous hypertensive rats	(102)
4	Soy protein	Asp-Leu-Pro	4.8 μM	HPLC, direct injection, and chromatographic isolation	–	–	(103)
		Asp-Gly	12.3 μM				
5	Tofuyo	Ile-Phe-Leu Trp-	44.8 μM	Gel filtration column chromatography and RP-HPLC	–	–	(85)
		Leu	29.9 μM				
6	Fermented soybean, Bacillus natto or subtilis	VAHINVGK	–	ACE inhibitory activity assay and simulated gastrointestinal digestion	–	–	(78, 104)
		YVWK					
7	Glycinin	SPYP	850 μM	Acid proteinase of *Monascus purpureus* (*In vitro* ACE inhibitory activity assay)	–	–	(62)
		WL	65 μM				
8	β-conglycinin	LAIPVNKP	70 μM	Acid proteinase of *Monascus purpureus* (*In vitro* ACE inhibitory activity assay)	–	–	(62)
		LPHF	670 μM				
9	Soy protein isolate digest	–	0.28 0.04 mg/mL	IEC, GF-FPLC, and IMAC (*In vitro* enzymatic digestion)	–	–	(60, 105)
10	Soybean protein isolated [Glycinin (A4 and A5)]	NWGPLV	21 μM	Electrospray ionization mass/mass spectrometry (MS/MS), gel filtration and reverse-phase chromatography, and solid phase	–	Spontaneous Hypertensive model rats	(64, 106)
11	Soy protein	YLAGNQ	14 mM	Pectin digestion	–		(53)
12	Douchi	–	–	Gel filtration chromatography	–	–	(82)
13	Glycinin	VLIVP	–	Protease P hydrolysis (*In vitro* ACE inhibitory activity assay)	–		(60)
14	Douchi	*Mucor*-type	0.204 mg/ml	Ultrafiltration, column chromatography, and RP-HPLC	–	–	(83)
		His-Leu-Pro	2.37 μM/L				
15	Protease (PROTIN SD-NY10) treated soy milk	FFYY	1.9 μM	Reversed-phase chromatography (ACE inhibitory activity assay)	–		(12, 71, 104)
		WHP	4.8 μM				
		FVP	10.1 μM				
		LHPGDAQR	10.3 μM				
		IAV	27 μM				
		VNP	32.5 μM				
		LEPP	100.1 μM				
		WNPR	880 μM				
16	Soy protein	IVF	5.4 μM	LC-MS/MS and QSAR	–	–	(65)
		LLF	6.7 μM				
		LNF	5.2 μM				
		LSW	3.4 μM				
		LEF	4.6 μM				
17	Fermented soybean meal	–	0.022 mg/ml	Ultrafiltration, gel chromatography, and RP-HPLC	–	–	(86)
18	Protein hydrolysate	HHL (primary peptide)	Highest Conc −983 μg/ml (IC_50_ = 224 ±13.1)	Spectroscopic determination of hippuric acid and using HPLC-DAD (diode array detection)	–	–	(23)
19	Soy protein	F2	17.2 μg/ml	Proteolytic cleavage using *Lactobacillus casei* spp. *pseudoplantarum* followed by fermentation	–	–	(107)
		F3 (fractions)	34.7 μg/ml				
20	Soy protein	LSW	2.7 μM	LC-MS/MS and QSAR	–	Vascular smooth muscle cells	(108)
21	Fermented soybean seasoning	SY	–	Reversed-phase chromatography	–	Spontaneously hypertensive rats	(104)
		GY					
22	Soybean protein	DMG	3.95 ±0.11 mM	*In silico* (virtual screening and docking)	S_1_ and S_2_	–	(6)
23	Tempeh	–	–	*In vitro* studies	–	–	(89)
24	Soy proteins	PPNNNPASPSFSSSS, GPKALPII, and IIRCTGC	–	–	–	–	
25	Soy protein isolate	IY	0.53 ±0.02 mM	Molecular docking	S_1_ and S_2_;S_1_, ${\text{S}}_{2^{\prime }}$ and Zn; S_1_; S_1_, ${\text{S}}_{2^{\prime }}$ and Zn	–	(20)
		YVVF	0.27 ±0.01 mM				
		LVF	0.36 ±0.01 mM				
		WMY	0.55 ±0.02 mM				
		LVLL	0.72 ±0.02 mM				
		FF	0.73 ±0.02 mM				
26	Soy iso-flavonoids	Genistein	634.96 (cACE) and 58.17 μM (nACE)	Molecular docking	S_1_ and S_2_; ${\text{S}}_{2^{\prime }}$ and ${\text{S}}_{1^{\prime }}$	–	(28)
27	Soybean fermented product (Chhurpi)	SVIKPPTDE	21.29 μM	Gastrointestinal digestion, molecular docking, and QSAR	–	–	(88)

^#^Subsites are shown in Figure 1.

The various soy proteins such as (proteins, protein isolates, and hydrolysates), soy foods (milk, sauce, paste, and other products), fermented products (fermented seasoning, tempeh, douchi, tofuyo, meal, extract, and chhurpi), and similarly, soy isoflavonoids showing the ACE inhibitory activities have been summarized below.

#### Soybean proteins

Zhao et al. (6) identified around 161 novel tripeptides using soybean hypothetical protein sequences (NCBI: KRH47534.1). Based on the toxicity and solubility studies, only 12 potential peptides (DTW, EGW, RPR, CIR, DMG, AGR, MDL, HDW, MDY, DVF, and LPR) were selected. ACE inhibitory activity analysis carried out using reversed-phase (RP)-HPLC showed that DMG, out of the 12 tripeptides selected was the most effective peptide with IC_50_ value of 3.95 ±0.11 mM. The docking revealed that the DMG peptide interaction with the active amino acids of the S_1_ and S_2_ subsites of the ACE (6).

Glycinin is obtained through enzymatic hydrolysis using protease P from the soybean and is considered a potential and potent ACE inhibitor. One such potent inhibitor includes protease P glycinin hydrolysate with sequence VLIVP (primary peptide). The inhibitor uses the peptic digestion of the soy protein the IC_50_ was found to be 14 μM for the peptide YLAGNQ (53, 60, 61). The peptides formed from the hydrolysis of β-conglycinin and glycinin through the acid proteinase of *Monascus purpureus* include SPYP (IC_50_ = 850 μM) and WL (IC_50_ = 65 μM) and LAIPVNKP (IC_50_ = 70 μM) and LPHF (IC_50_ = 670 μM), respectively (61, 62). Furthermore, using the Edman’s process and peptic digestion of the soybean protein hydrolysates, many ACE inhibitors were identified which include IA, YLAGNQ, FFL, IYLL, and VMDKPQG having IC_50_ values 153, 14, 37, 42, and 39 μM, respectively (61, 63).

Soybean protein isolate (SPI) is an abundant low-cost protein source and was known to possess many inhibitory peptides from SPI hydrolysates namely DLP, LSW, DG, and NWGPLV (IC_50_ = 21 μM) (20). Around eight novel inhibitory peptides were identified and among them, NWGPLV was found to be the most potent peptide which was treated with D3 protease obtained from soybean (64). Using the approach of LC-MS/MS along with the QSAR model, soybean protein hydrolysate treated with pepsin and thermolysin yielded IC_50_ values 51.8 and 53.6 μg/mL, respectively, and also identified five novel tripeptides having potential inhibitory activities (shown in Table 1) (65). Many di and tripeptides were enzymatically isolated using trypsin, pepsin, and thermolysin from soybean protein and calculated IC_50_ values of 33 such peptides (65). Xu et al. (20) have identified many novel peptides with inhibitory activities against ACE. Peptides with good results include LVF, WMY, IY, FF, YVVF, WMY, and LVLL with significant hydrophobic and predicted activity scores. IY binding with the active sites and occurring in subsites S_1_ and S_2_ and WMY (S_1_, ${\text{S}}_{2^{\prime }}$ and interacting with Zn) were found to be potent inhibitors for the enzyme. YVVF and LVLL occur in the subsites S_1_ and ${\text{S}}_{2^{\prime }}$ and form hydrogen bonds with the catalytic site zinc ion. whereas LVF occurs in S_1_ (20). Although, Rudolph et al. (66) identified many ACE inhibitors including IY with IC_50_ = 5.2 ±1.4 μM/L which was higher than that shown by Xu et al. (20). The soy peptides also show anticancerous activities (67) and may reduce many severe physiological (age-dependent) diseases (68).

*Soy protein hydrolysates* (SPHs)—The SPHs are obtained through a sequential processing of soy proteins with different methods (hydrolysis, thermal treatment, gastrointestinal digestion, and microbial fermentation) yielding a mixture of peptides (69). The soy proteins have shown good inhibitory activities and are widely being used as potential functional foods which can be commercialized to use for ACE inhibition. Studies done by Daliri et al. (70) have reported ACE inhibitory activity using soybean protein hydrolysates (70) and similarly, Bollati et al. (25) reported ACE activity with an IC_50_ = 0.33 0.01 mg/ml (25).

#### Soyfoods

*Processed soy milk*—The PSM was used for the identification of ACE inhibitory peptides. It was digested by the bacterial proteases and obtained eight novel peptides showing activity against the enzyme ACE. Among them, two peptides namely, FFYY and WHP found to be more suitable than others, and therefore, PSM could act as a good source for the development of antihypertensive drugs and it is, in turn, a suitable candidate food (71). A similar study carried out by Shimakage et al. (12) who identified eight novel peptides including FFYY, WHP, FVP, LHPGDAER, IAV, VNP, LGPP, and WNPR with IC_50_ 1.9, 4.8, 10.1, 10.3, 27.0, 32.5, 100.1, and 880.1 μM possess inhibitory activity against the ACE. It was observed that FFYY and WHP were more potent inhibitors than others (12).

*Soy sauce*—Fermented soy sauce using the Japanese method was used to extract antihypertensive peptides. Two fractions were obtained as high (Hw) and low (Lw) molecular weight using gel filtration chromatography. It was observed that the Hw fraction showed inhibitory properties when orally given to rats (72).

*Soy paste*—Fermented soybean paste was used for the identification of ACE inhibitors. The *in vitro* analysis showed that Korean soy paste can inhibit ACE. A novel peptide was isolated and identified with IC_50_ 2.2 μg/ml for HHL (73). Li et al. showed the similar reports (74).

Other soy products (milk, yogurt, and natto)—Other works carried out using raw, steamed, and soaked soybean and different natto samples revealed they can be potential functional foods and showed ACE inhibitory activity (75). Soy food products including natto, soy yogurt, soymilk, tempeh, and tofu showed inhibitory activity against ACE after *in vitro* gastrointestinal digestion while traditional soymilk both raw and cooked showed the highest antihypertensive inhibitory activity. Among fermented soy foods, tempeh showed the least inhibitory activity than natto and soy yogurt which depicted higher inhibition. It also reported that two major proteins namely 7S and 11S of soybean also showed effective ACE inhibition (7). A Japanese traditional fermented food, natto is prepared by fermenting it with boiled soybeans and *Bacillus natto* and found to be effective against hypercholesterolemia, arteriosclerosis, and hypertension. Previous studies showed that spontaneous hypertension rats (SHRs) were fed with not boiled natto showed decreased blood pressure while Okamoto et al. (76) showed that the ACE inhibitory potential is gained through boiling and fermentation processes. Fermented milk is also known to possess inhibitory activities against the ACE, the studies carried out by Fan et al. (77), Hernández-Ledesma et al. (78), and (77–79).

#### Fermented soy products

*Fermented soybean seasoning (FSS) or Soy sauce-like seasoning*—The FSS was modified from the soy sauce’s normal production. It was more concentrated than that of normal sauce and contain 2.7 folds more peptides and in terms of inhibition IC_50_ for FSS found to be 454 μM and regular soy sauce IC_50_ was 1,620 μM. The peptides isolated were AW IC_50_ = 10 μM, GW IC_50_ = 30 μM, AY IC_50_ 48 μM, SY IC_50_ 67 μM, GY IC_50_ = 97 μM, AF IC_50_ = 190 μM, VP IC_50_ = 480 μM, AI IC_50_ = 690 μM, VG IC_50_ = 1,100 μM, and nicotianamine IC_50_ = 0.26 μM (80). The potent peptides among these were found to be GY and SY in the rat models used (81).

*Douchi*—Douchi is a Chinese recipe made of fermented soybean and is in various traditional medicines. Soy paste and sauce have also been prepared from douchi. Douchi was fermented along with *Aspergillus egypticus* and the peptides were analyzed thereon. It showed better results against ACE inhibition (82). Another study showed that *Mucor*-type douchi (Yongchuan douchi), one of the three types of douchi prepared in China with IC_50_ value 0.204 mg/ml. The peptide which was isolated was found to be HLP (His-Leu-Pro) with a 50% inhibitory concentration of 2.37 μmol/L (83). Similar results were reported by Li et al. (84).

*Tofuyo*—It is a traditional Chinese fermented prepared from tofu similar to that of cream cheese. IFL and YL were isolated from the tofuyo using gel filtration and RP HPLC methods with IC_50_ 44.8 and 29.9 μM, respectively (85).

*Fermented soybean meal*—Using the *Bacillus subtilis* natto, fermentation and proteolysis were carried out. The inhibitory activity was found to be 84.1% with IC_50_ value of 0.022 mg/ml (86).

*Fermented soybean extract*—The composition of the inhibitory peptide is LVQGS, isolated using the Edman degradation method with IC_50_ value 22 μg/mL while the inhibitory activity of the fermented extract was obtained as 1.46 mg/ml (87).

*Chhurpi*—Soy *chhurpi* is a product prepared using the fermented soymilk and proteolytic *Lactobacillus delbrueckii* WS4. With the help of gastrointestinal digestion, molecular docking, and QSAR, a glycinin-derived peptide was identified, SVIKPPTDE with an IC_50_ value of 21.29 μM and reported the first production of *chhurpi* soy cheese (88).

*Tempeh*—It is an Indonesian dish made from fermented soybean alongside *Rhizopus* sp. It is shown to possess health-promoting benefits for humans. Tempeh inhibits angiogenesis during cancer, improves bone’s health, and acts as an antioxidant and anti-bacterial agent. It is also useful in treating Alzheimer’s disease and dementia. Chalid et al. through *in vitro* analysis showed that tempeh has inhibitory activity (89). Tempeh derived-isoflavonoid, genistein showed anti-angiogenesis properties (89, 90).

#### Soy isoflavonoids

Flavonoids are polyphenolic secondary metabolites primarily found in plants and some bacteria. They play a wide variety of functions in plants from signaling molecules, phytoalexins, detoxifying agents, stimulating germination of spores and seeds and acting as attractants of pollinators, and many others. One of the three categories of flavonoids include isoflavonoids (91). Recently, to identify natural compounds which could act as inhibitors of ACE plant-derived polyphenolics, peptides, and terpenes are being explored owing to their pharmacological properties (92) including isoflavonoids. Isoflavones are abundant in soybean and act as health enhancers. Daidzein, Glycitein, and Genistein are the primary isoflavones in soybean. They help in the prevention of cancers, reduce the level of cholesterol, and lower hypertension (93). Previous studies carried out on soybean isoflavonoids (94–97) showed that these isoflavonoids have inhibitory action against ACE and aid in protection from renal diseases (98). The soy isoflavones resemble mammalian estrogen (phytoestrogen). Ramlal et al. (28) have shown the molecular basis of the selectivity of isoflavones from soybean namely Genistein, Glycitein, and Daidzein as ACE inhibitors through *in silico* molecular docking approaches. According to the study, Genistein was found to be more potent as compared to the others having more hydrogen bonds and hydrophobic interactions with the catalytic subsites ensuring a tight binding which is further correlated with the observed inhibition constants. It was observed that Genistein is a moderate cACE but selective nACE inhibitor (inhibition constants 634.96 and 58.17 μM, respectively) while the other two isoflavones Daidzein and Glycitein exhibited selective inhibition profiles for the N domain of ACE (inhibition constants 47.37 and 228.5 μM, respectively) (28).

## Discussion

Although, the common medication for the treatment of CVDs and renal diseases including heart failures and hypertension the commercial drugs are preferred. However, despite the lower rates of success due to prolonged treatment procedures, persistent side effects (angioedema, cough), and no one-time remedy of the commercially available drugs (Enalapril, ramipril, and similarly captopril, perindopril, and lisinopril) are recommended for the initial therapy (15). Furthermore, with the outbreak of coronavirus, it has been shown that the virus uses ACE II as its receptor to invade the cells (99). Moreover, since the outbreak of the pandemic and even before the occurrence, herbal medicines were the preferred choice over synthetic drugs due to their side effects (100, 101). Therefore, phytocompounds are being searched for their inhibitory activity against ACE as an alternative therapy. This article describes and highlights the various ACE inhibitory obtained from proteins and isoflavonoids from soybean signifying the importance in the treatment of hypertension and heart-related problems for making future drugs.

## Conclusion and future prospects

Angiotensin-converting enzyme is a key enzyme in the RAAS which helps in the regulation of hypertension. The overproduction of angiotensin by the activity of ACE leads to a medical condition known as hypertension and also due to the consumption of synthetic drugs which lead to cause side effects and sometimes even death. Therefore, it becomes very important to control or inhibit the ACE to control/treat hypertension using the phytocompounds like saponins, terpenes, and isoflavonoids. The clinical and therapeutic importance of ACE inhibitors is well understood. The identification of phytocompounds with potential ACE inhibitor activity can be good alternative for chemical drugs because of no or minimum side effects than the latter ones. The article provides a clue to researchers like plant breeders who can breed and develop specialty soybean varieties meant to provide ACE inhibiting compounds. It also provides a hint to the pharmacy sector to capitalize the soybean phytocompounds as ACE inhibitors in place of synthetic drugs. Therefore, natural compounds, and other phytoconstituents should be searched for their inhibitory activity against ACE for a safer alternative and future drug design.

## Author contributions

AyR and AmR contributed to the conception and design of the study. AyR wrote the first draft and curated the data. All authors equally contributed to the manuscript revision, editing, APC, read, and approved the published version.
